# Aspergillosis in Wild Birds

**DOI:** 10.3390/jof7030241

**Published:** 2021-03-23

**Authors:** Pascal Arné, Veronica Risco-Castillo, Grégory Jouvion, Cécile Le Barzic, Jacques Guillot

**Affiliations:** 1 Ecole Nationale Vétérinaire d’Alfort, Centre Hospitalier Universitaire Vétérinaire de la Faune Sauvage (Chuv-FS), 94700 Maisons-Alfort, France; veronica.risco-castillo@vet-alfort.fr (V.R.-C.); cecile.le-barzic@vet-alfort.fr (C.L.B.); 2 Ecole Nationale Vétérinaire d’Alfort, Dynamic Research Group UPEC, EnvA, USC Anses, 94700 Maisons-Alfort, France; gregory.jouvion@vet-alfort.fr (G.J.); jacques.guillot@vet-alfort.fr (J.G.); 3 Ecole Nationale Vétérinaire d’Alfort, Biopôle Alfort, 94700 Maisons-Alfort, France

**Keywords:** aspergillosis, wildlife, bird, *Aspergillus*

## Abstract

The ubiquitous fungi belonging to the genus *Aspergillus* are able to proliferate in a large number of environments on organic substrates. The spores of these opportunistic pathogens, when inhaled, can cause serious and often fatal infections in a wide variety of captive and free-roaming wild birds. The relative importance of innate immunity and the level of exposure in the development of the disease can vary considerably between avian species and epidemiological situations. Given the low efficacy of therapeutic treatments, it is essential that breeders or avian practitioners know the conditions that favor the emergence of Aspergillosis in order to put adequate preventive measures in place.

## 1. Introduction

Aspergillosis holds a very special place in veterinary and human medicine, because it is the main type of mycosis affecting birds and mammals, including human beings [1]. The Aves class is particularly concerned, due to [2,3,4]. The great diversity of species susceptible to this infection, including domestic and wild animals living in captivity or in natural environments; the omnipresence of the *Aspergillus* fungi in varied indoor and outdoor environments on all continents except Antarctica; the ability of this opportunistic mold to grow efficiently in its ecological niche and in birds following inhalation of its spores; the severity of the infections observed, which can result in a high mortality rate; the fact that, despite the relatively large number of case reports and amount of experimental data, particularly those obtained from poultry species, the pathogenesis of avian aspergillosis is still poorly understood [2,5].

Historically, the birds’ contribution to our scientific knowledge of fungal diseases in animals is far from negligible (Table 1). Since the first reports in the early 1800s of a “mold or blue mucor” in a greater scaup (*Aythya marila*) by Montague and in an Eurasian jay (*Garrulus glandarius*) by Mayer and Emmet, many observations based on either fungal microscopic morphology or descriptions of lesions have been made on specimens of various avian species. Bronchi, lungs, air sacs, and, secondarily, bones are the most affected organs. Rayer and Montagne identified *A. candidus* from the air sacs of a bullfinch (*Pyrrhula pyrrhula*) and recognized, for the first time, the association of *Aspergillus* fungi with typical lesions. Following the identification of other *Aspergillus* species (Table 1), the first description of *A. fumigatus* occurred as a filamentous fungus exhibiting a “smoky grey color” in a wet mount. It was isolated in the bronchi and air sacs of a great bustard (*Otis tarda*) from the zoological park of Frankfurt [6,7].

In young birds, the disease referred to as “brooder pneumonia” is the most commonly diagnosed infection in nestlings around the time of hatching. Other synonyms for avian aspergillosis include fungal or mycotic pneumonia, pneumomycosis, bronchomycosis, and colloquialisms such as “asper” and “air sac” [3].

The objective of this review was to synthesize current knowledge related to aspergillosis in wild avifauna and pet or game birds that could be useful to all those involved in the management of these kinds of animals (veterinarians, zookeepers, pet owners, conservation program managers, wildlife rehabilitators).

## 2. Etiology and Ecology

Only a small percentage of the approximately 340 accepted *Aspergillus* species are implicated in the development of avian aspergillosis [8]. *Aspergillus fumigatus* is by far the most prevalent species, representing up to 95% of cases, in both wild and domestic avifauna [3,9]. Isolation of *A. flavus* is less common and has been described in falcons [10,11], parrots [12], and a royal tern *Thalasseus maximus* [13]. The species *A. niger* has been isolated from sick ostriches (*Struthio camelus*) [14], falcons [10,15,16], a Eurasian eagle owl (*Bubo bubo*) [17], and African grey parrots (*Psittacus erithacus*) [18]. The species *A. terreus* [16,19,20], *A. versicolor* [21,22], *A. oryzae* [23,24], *A. nidulans* [25,26], *A. amstelodami, A. glaucus*, and *A. nigrescens* [27] have been unfrequently reported in diseased birds. Mixed infections with *A. flavus* and *A. niger*, *A. fumigatus* and *A. niger*, and *A. fumigatus* and *A. oryae*, have been described in captive ostriches, falcons, and parrots, respectively [10,23,28]. Concomitant infections of the anterior parts of the eye in lovebirds [29] and of the lungs in white storks [30] were found to result from the association of *Aspergillus* sp. and *Candida albicans* with different zygomycetes. Although cryptic *Aspergillus* species may account for 10 to 14% of all clinical strains in human patients [31,32], data remain scarce in the veterinary field, and related studies have used relatively small samples. By sequencing the *β-tubulin* gene (*benA*), Vedova et al. [33] identified *A. sydowii* for the first time in a Swinhoe’s *pheasant (Lophura swinhoii*). Using the same target, Talbot et al. [26] reported the new species *A. restrictus* among 26 avian clinical isolates obtained from a Java finch with disseminated invasive aspergillosis. *A. allahabadii*, an unknown species in birds, was cultured from the air sac of a cormorant used for traditional fishing and finally identified by the partial *benA* gene and ITS sequencing [34]. Molecular characterization (partial *benA* and *camA* genes sequencing) of two *Aspergillus* section *Fumigati* and one *Aspergillus* section *Flavi* [35] isolated from the black-browed albatross *(Thalassarche melanophris)* conclusively showed the presence of the two first isolates (*A. fumigatus stricto sensu*) but not the last one (*A. flavus/oryzae* lineage). The same team [36] identified *A. fumigatus ss* from lesions of three other free-ranging aquatic birds: the white-chinned petrel (*Procellaria aequinoctialis*), the neotropical cormorant (*Nannopterum brasilianus*), and the brown-hooded gull (*Chroicocephalus maculipennis*). Following the amplification of the *β-tubulin* and *rodlet A* (*rodA*) genes, the 53 isolates tested by Spanamberg et al. [37] were confirmed as being *A. fumigatus stricto sensu.* No cryptic species from the *Fumigati* section were detected among 43 clinical and 34 environmental “*Aspergillus fumigatus*” samples in Californian rehabilitation centers using *β-tubulin* and *camA* gene sequencing [38]. The screening of 159 independent isolates from Germany by *camA* sequencing led to similar conclusions [39]. Those results support the preponderant role of *A. fumigatus stricto sensu* in the development of avian aspergillosis.

Indirect enzyme-linked immunosorbent assay (ELISA) tests on penguins have shown a seropositivity rate of 94% in a captive population, with an average of 60% of birds from wild colonies producing anti-*Aspergillus* spp. IgGs [40,41]. As evidenced by air samples, a fungal load is regularly detected in outdoor [42,43] or indoor environments where birds may be found. Such observations have been done in wildlife rehabilitation centers [43,44] and zoological aviaries [45,46,47,48,49,50] sheltering various species. Air contamination levels and the mycobiota composition are characterized by dynamic or cyclic variations [45,46,47,49]. These fluctuations may be related to season, environmental management, or to the presence of natural soil, plants, or litter [45,48]. A strong correlation between litter fungal contamination and aerial mycobiota in poultry houses corroborates the aerosolization of fungi found in litter and indicates that organic bedding may constitute the main reservoir of indoor contamination [51,52]. *Aspergillus* fungi may also be found in water and dust [43,53]. It is noteworthy that nests of wetland birds or nest-boxes of passerines can provide reservoirs of pathogenic fungi like *A. fumigatus* or *A. flavus* with up to 650 colony-forming units (CFU)/g of dry mass of the nest material [54,55]. *A. fumigatus* has been isolated on the feathers of 30% [56] of free-living house sparrows (*Passer domesticus*) and on 6 to 13% of pharyngeal/tracheal swabs sampled from captured pink-footed geese (*Anser brachyrhynchus*), Canada geese (*Branta canadensis*), or herring gulls (*Larus argentatus*) that presented as healthy carriers [57]. Positive tracheal samples from seabirds undergoing rehabilitation [43] or trapped hatchlings to second-year goshawks (*Accipiter gentilis*) were not rare and might indicate either exposure to the fungus or true sickness [19].

## 3. Geographic Distribution and Seasonality

With the exception of Antarctica, aspergillosis in wild avifauna has been reported on all continents [2]. By testing eight populations of four different Spheniscidae species for plasma *Aspergillus* IgG by ELISA, Graczyck and Cockrem [40] observed a latitude-related decrease of antibody seroprevalence, which seemed to reflect a decrease in the exposure of subantarctical to antarctical penguins to *Aspergillus* spp.

**Figure 1:**
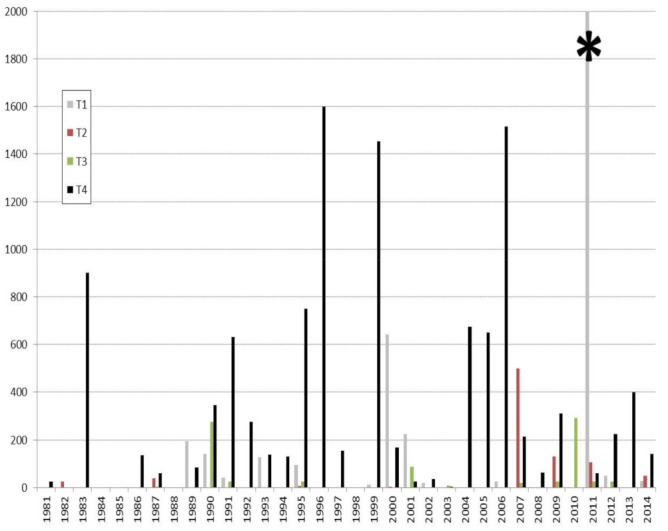
Quarterly die-off reports attributed to Aspergillosis in North-American avifauna. (USGS 1981–2014). T1: January to March; T2: April to June; T3: July to September; T4: October to December. * To improve the clarity of the diagram, an episode that occurred in 2011 associated with a very high mortality rate (7000 dead) is not represented in full.

Most Aspergillosis outbreaks in North-American waterfowl population happen in fall or early winter, although individual cases can occur at any time (Figure 1) [58,59,60,61,62,63].

This apparent seasonality may result from how local climatic conditions and fungal ecology interplay or integrate a bird’s intrinsic factors, such a particular level of susceptibility at that moment of the year. Ambient temperatures and humidity play important roles in the lifecycles of fungi and the level of a bird’s exposure to fungal spores, as can be seen inside poultry houses. *Aspergillus* fungi multiply during the wet period, producing abundant xerophilic spores which are then dispersed into the atmosphere when conditions become dry [5,64]. Higher morbidity and mortality rates have thus been observed in red-vented cockatoos (*Cacatua haematuropygia*) during the monsoon season [65].

**Figure 2:**
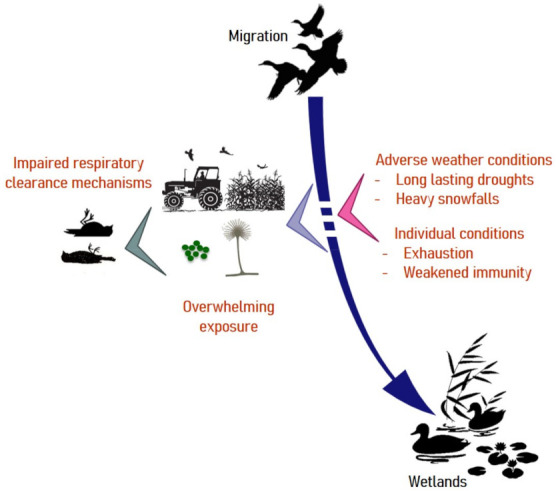
Risk factors associated with aspergillosis in migratory birds. Under usual climatic conditions, waterfowl exploit wetlands for food. Weather disturbances can force birds to take refuge on cultivated lands and feed on crop residues that are sometimes heavily contaminated by molds. Under such circumstances, exhausted individuals may suffer from airway clearance dysfunction and develop aspergillosis with a fatal outcome.

As summarized in Figure 2, mallards, which appear to be highly susceptible to Aspergillosis may resort to rotting agricultural waste during inclement weather. Weakened by forced displacements in search for food, they may land on contaminated crops. The timing and locations of these few-days lasting outbreaks are highly suggestive of a common source of conidia and exposure within a limited time frame [27], as has been reported in poultry [66].

## 4. Host Range and Impact

Data on the frequency of aspergillosis development in wild birds is still fragmentary and may be partially biased by studies targeting specific birds or particular geographical areas. Scientific data relative to free-ranging or captive wild birds gather descriptions of sporadic cases, outbreaks and results obtained [67] during active or passive surveillance operations [2,68]. When die-offs occur, a highly variable fraction of carcasses or moribund birds is generally submitted to post-mortem extensive investigations. Furthermore, means engaged to establish the final diagnostic are sometimes limited to the observation of gross lesions [67] and sometimes histopathology [69,70,71] or based on phenotypical or polyphasic characterization of the isolated fungi [30,35]. Therefore, evaluations of the prevalence of aspergillosis may be overestimated and should instead refer to a “mycotic disease” when conclusions result solely from the observation of “typical lesions” [30,72].

A compilation of epizootics or mortality surveys in wild birds has been published by Converse [2]. Species that are mostly represented belong to the following taxonomic orders: Anseriformes (swans, geese and ducks), Accipitriformes (eagles and hawks), Charadriiformes (shorebirds and gulls), Passeriformes (singing birds), and Galliformes (fowls, quails and pheasants). In North America, aspergillosis is considered a common disease in waterfowl, gulls, and crows and occasionally in other songbirds and upland gamebirds [27]. Early publications mentioned the role of aspergillosis in the death of 180 wood ducks (*Aix sponsa*) in Illinois [58], 170 [60], 270 [62] and more than 1500 [73] mallards (*Anas platyrhynchos*) in three distinct episodes, as well as 2000 Canada geese (*Branta canadensis*) in Missouri [59] and 1000 to 1500 common crows (*Corvus brachyrhynchos*) in Nebraska [61]. Periodic reports published by the National Wildlife Health Laboratory of the United States Geological Survey indicate that aspergillosis is regularly associated with rapid die-offs among North American avifauna [74]. Mortality rates oscillate between tens, hundreds, and sometimes thousands of casualties (Figure 1). Dead and moribund birds belonging to one or several species are often found in direct proximity to moldy grain sources or rotten ensilages [58,73,74]. Waterfowl (mallards, geese) and gulls (*Herring gulls*) are the most commonly affected species, although their flocking behaviour and size may increase the likelihood of outbreak detection [74]. A Spanish serological survey detected the highest percentages (1.7 to 3.1%) of positive birds in grey herons (*Ardea cinerea*), mallards, and coots (*Fulica atra*) living in the Guadalquivir marshes [75]. *Aspergillus* antibodies and antigens were detected in 9–50% and 27–31% of nocturnal heron chicks (*Nycticorax nycticorax*) belonging to distinct populations of New York Harbor estuary, respectively [76].

In the framework of mortality studies, systematic postmortem evaluations have been made in different targeted wild species, either emblematic or those proposed as potential indicators of aquatic health, such as swans or loons. Close inspection of a series of carcasses collected during pluri-annual surveys allowed a better appreciation of the impact of “aspergillosis” on avifauna (Table 2). This fungal disease was recognized as the primary cause of death for 6 to 23% common loons [72,77,78,79] and 4 to 21% swans [67,69,71,80]. During a two-year study on morbidity and mortality in a Wildlife refuge in New York [81], aspergillosis was diagnosed in 31% of necropsied birds, mostly herring gulls. Olias et al. [30] examined 101 dead white stork chicks coming from 10 different German regions and found 45 cases of invasive fungal pneumonia, including 22 that were directly attributable to *A. fumigatus*. Quite similar aspergillosis-related rates of mortality (Table 2) were found for the Eurasian crane [82] and the threatened Whooping crane [70]. A retrospective analysis of aquatic bird mortality events in the US between 1971 and 2005 revealed that fungal causes, mainly due to *Aspergillus* fungi, accounted for 7% of mortalities attributed to infectious diseases [76].

Occasional reports of mortality have involved conservation-dependent or critically endangered species like California condors (*Gymnogyps californius*) (Rideout et al. 2012), Hawaiian geese (*Branta sandvicensis*) [83], brown kiwis (*Apteryx mantelli*) [48], helmeted honeyeaters (*Lichenostomus melanops cassidix*) [84], and yellowheads (*Mohoua ochrocephala*) [85]. Recently, aspergillosis killed seven kakapo parrots (*Strigops habroptila*), a species that has fewer than 150 fully grown birds left in the world [86]. Aspergillosis is a potentially important cause of failure of conservation and translocation programs of avian species, as highlighted by the example of the very rare New-Zealand endemic yellowhead [85] and stichbird (*Notiomystis cincta*). Aspergillosis was identified as the cause of the death in 11/31 free-ranging stichbirds during a 15-year period [87], in 6/9 adults between 1995 and 1997 in another location [88] and has driven the removal of a released population from their new environment due to excessive mortality [89].

While Anseriforms appear to be highly susceptible to the disease in both free-living and captive conditions [64,90,91,92], wide variations of prevalence do exist for other avian orders. Several case reports refer to sporadic aspergillosis in free-ranging birds of prey [19,93,94,95,96,97,98], but this mycosis is less often diagnosed in this category in comparison with captive or recently-captured free-ranging raptors, for which it is the most important non-traumatic pathology [15,99,100]. Many descriptions of cases concern various species of captive parrots [12,23,65,101,102,103,104,105,106,107]. The high frequency of reports on Accipitriformes, Falconiformes, and Psittaciformes is probably due, in part, to their popularity as appreciated pets or falconry birds. As underlined by the scientific literature, aspergillosis is a major concern for veterinarians and zookeepers in populations of penguins under human management, but it is rarely observed in free-ranging populations [108].

Death by aspergillosis has been described in captive gentoo penguins [115], Magellanic penguins [29,116,119,120], Humboldt penguins (*Spheniscus humboldti*) [121], king penguins (*Aptenodytes patagonica*) [41], and African penguins (*Spheniscus demersus*) [122,123]. Postmortem diagnosis confirmed the implication of aspergillosis in 29%, 33%, and 48% of serial deaths in Magellanic penguins, king penguins, and gentoo penguins, respectively [41,115,124].

## 5. Disease Predisposition

In domestic birds, both field data and experimental results have clearly demonstrated a higher susceptibility of turkeys (*Meleagris gallopavo*) and quails (*Coturnix japonica*) to aspergillosis when compared to chickens, for example [125]. Furthermore, differences in susceptibility have been demonstrated between different turkey and chicken lineages following experimental inoculation of spores [3]. In wild species, empirical data claim that gyrfalcons (*Falco rusticolus*) and hybrids, merlins (*Falco columbarius*), goshawks, red-tailed hawks (*Buteo jamaicensis*), ospreys (*Pandion haliaetus),* rough-legged hawks (*Buteo lagopus*), golden eagles and snowy owls (*Nyctea scandiaca*) are highly susceptible to aspergillosis [99,126]. Similar observations have been made for the blue-fronted amazon (*Amazona aestiva*), the African grey parrot, and pionus parrots (*Pionus* spp.) among psittacine birds [4]. When mixed groups experience aspergillosis, mortality may affect only some species. In an American aquarium, 19% of 85 tufted penguins (*Lunda cirrhata*) and 30% of 20 pigeon guillemots (*Cepphus columba*) died, but none of the 20 rhinoceros auklets (*Cerorhinca monocerata*), despite being housed in the same area [127]. In Cheasapeake bay, 36 of 50 canvasbacks (*Aythya valisineria*) succumbed to aspergillosis, but 12 redheads (*Aythya americana*) and 12 scaup ducks (*Aythya* sp.) which belonged to the same captive flock were unaffected [90].

Souza and Degernes [80] found than male swans were twice as likely as females to have fungal disease, whereas the role of gender in susceptibility to aspergillosis has not been documented in raptors [126]. Cumulative data show that young animals are particularly prone to the development of aspergillosis in poultry [3] and wild birds. Young raptors, notably, immature red-tailed hawks, seem to be more susceptible between 2 to 4 months of age [99,126]. Aspergillosis is prominent in captive downies and juveniles (Table 2) of different waterfowl species (northern geese and perching ducks) and in rehabilitated juvenile penguins in comparison to adults [91,92,120]. Immature loons were found to be significantly more likely to be affected than breeding or wintering adults [72]. A similar higher incidence among juveniles has been noted in free-ranging swans [69] that has been associated with the occurrence of more severe lesions than in adults or sub-adults [80]. During a 2-year survey, 96% of the herring gulls diagnosed as having aspergillosis were sub-adult birds [81].

There are multiple external factors implicated in causality. Accidental ingestion of heavy metals, particularly lead, has been associated with aspergillosis in loons [78] and swans [71]. However, Souza and Degernes [80] established that lead exposure is not a risk factor for the development of the mycosis, which remains mild in affected swans. These observations indicate that rapid death following primary acute lead intoxication might prevent the development of severe fungal lesions. Two migrating Eurasian black vultures (*Aegypius monachus*) were found to be suffering from aspergillosis and acute carbofuran insecticide poisoning (Jung et al. 2009). Other non-infectious conditions associated with aspergillosis in wild avifauna include trauma, gunshots, extreme wear to flight feathers, oiling, emaciation, and exhaustion consecutive to migration [19,59,72,78,97,109]. Mixed infections are not uncommon in free-living and captive wild birds. They do not always indicate whether aspergillosis is a primary or secondary infection. Reported intercurrent diseases include tuberculosis in birds of prey and in an egret *Egretta thula* [25,94,128], salmonellosis in loons [109], polymicrobial infection in Cape vultures (*Gyps coprotheres*) [129], botulism in shore birds [81], psittacine beak and feather disease and Budgerigar fledging disease in an African grey parrot [130], Eastern equine encephalitis in African penguins [122], hepatitis E virus infection in Himalayan griffons (*Gyps himalayensis*) [21], Pox virus infection in a royal tern (*Thalasseus maximus*) [13], avian malaria in Magellanic penguins [116,131], helminthosis in a black-eared milan (*Milvus migrans*) [98], a blue jay (*Cyanocitta cristata*) [132] or a herring gull [133], tracheal trematodosis in swans [71], sarcocystosis in parrots [104], trichomonosis in raptors [134,135], and amebiasis in great blue turacos (*Corythaeola cistata*) [136]. The use of broad-spectrum antibiotics and immunosuppressive therapies (corticosteroids) can stimulate the occurrence of fungal pathogens in debilitated birds [23,107,137].

Concomitant primary pathogenic agents and/or diseases were identified in 64 of 94 *Aspergillus*-positive cases in captive falcons. When compared with a control group of 2000 disease-free falcons, *Babesia shortii, Mannheimia haemolytica, Escherichia coli*, and Falcon herpesvirus infection, but not *Trichomonas* infection, appeared to be suitable candidates as predisposing factors for aspergillosis [10].

As emphasized by observations in birds of prey and penguins, captivity itself may be a major contributor to the emergence of aspergillosis, especially when deleterious conditions such as overcrowding, poor ventilation, thermal discomfort, or a high level of exposure to respiratory irritants (ammoniac, dust) occur [99,102,107,126]. This is even truer in the case of animals recently captured in the wild. Increased incidences of fungal infections have been registered for parrots, raptors, and penguins coming into permanent or transitory captivity [23,116,135]. Aspergillosis was diagnosed in 31 out of 42 Philippine red-vented cockatoos and 55 out of 179 houbara bustards (*Chlamydotis undulata macqueeni*) after they were trapped in the framework of legal or illegal trades [65,138]. In companion animals, hypovitaminosis A [101], participation in a bird song contest [37], transportation [139], intensive falconry training [126], or a change of ownership [99] have been identified as potential stressors eliciting aspergillosis.

Aspergillosis is a common and particularly feared complication of oil spills in rehabilitation centers with high mortality rates occurring on either oiled and non-oiled common murres (*Uria aalge*), razorbills (*Alca torda*), common loons, New Zealand dotterels (*Charadrius obscurus aquilonius*), and Magellanic penguins [120,140,141,142,143].

## 6. Pathogenesis

Aspergillosis typically occurs after the inhalation of the ubiquitously available spores, but localized infections of the eye or the skin are possible [29,144,145]. Several forms are classically recognized. Acute disease may occur following exposure to an overwhelming number of spores from a point source. More chronic forms are slowly progressing infections that affect birds showing some degree of immunodeficiency and may result from the regular inhalation of spores. Understanding the relative contributions of the level of exposure and the transient susceptibility of the host in the initiation of infection remains a challenge [3].

The question surrounding the infectious dose is still fundamental. Experimental models of acute aspergillosis indicate that unless the dose is massive (10^6^ to 10^8^ spores/bird), exposure via different ways rarely produces disease and may affect species differently [5]. The intra-tracheal administration (IT) of 1.35 × 10^6^ spores/starling (*Sturnus vulgaris*) induces mortality in 100% of affected individuals in 6 days [146]. Inoculum of 10^7^ (IT) and 2.10^7^ spores (injected into the thoracic air sac) killed 2/5 and 4/4 rock pigeons *(Columba livia*), respectively, but none of five gyr-saker hybrid falcons (*Falco rusticolus x F. cherrug*) submitted to a lower IT dose died [147,148]. In another experiment, all falcons (two peregrine falcons *Falco peregrinus* and two saker x gyrfalcons) receiving 2.10^7^ spores IT succumbed to the infection following very rapid health deterioration [149]. A lethal dose 50 (LD50) of 12.03 × 10^6^/bird was established in Japanese quails by Chaudhari and Sadana [150]. Based on histopathological results as definitive diagnosis of experimental aspergillosis in gyr-saker hybrid falcons, Fischer et al. (2018) calculated different minimal infectious doses (MID_10 (±S.E.)  _=_ _ 10^1.95^ ^±^ ^1.26^, MID_50 (±S.E.) _ =_ _ 10^4.52^ ^±^ ^0.67^, and MID_90 (±S.E.) _ =_ _ 10^7.10^ ^±^ ^1.33^). Based on MID_10_ and on respiratory minute ventilation, the authors extrapolated their data to determine the tolerable spore concentration in ambient air that must be inhaled over 24 h by a resting juvenile falcon as being 86.50 CFU/m^3^/h. Air samples collected under various contexts linked to birds have highlighted the wide variability of aerial fungal loads [5,45,46,47,49]. Maximal indoor measurements of the airborne *A. fumigatus* spores concentration in a German falcon breeder center [151], three Californian rehabilitation centers and one Italian rehabilitation center all sheltering aspergillosis-diseased birds were 45, 50, and up to 525 CFU/m^3^, respectively. The difficulty in clearly correlating exposure to given fungal loads and aspergillosis occurrence combined with the variability of experimental results underline the preponderant role of spontaneous susceptibility in birds, which differs at the species, age, and even individual level. In fact, most mycoses in captive birds could be related to issues of husbandry-associated stress, rather than to the presence of specific fungal populations in the air [47].

The anatomo-physiological characteristics of the respiratory systems of birds as well as their innate immune responses may suggest that all avian species are susceptible to developing aspergillosis under favorable circumstances. Spores of *A. fumigatus,* when inhaled through the nares, are small enough [9,152] to bypass mucociliary-dependent clearance by the upper airways and disseminate first in the posterior air sacs rather than in the cranial ones, in accordance with the gas pathway through the lungs [153,154]. The trachea or syrinx may be involved as well due to anatomical particularities, such as tracheal loops in swans [80] or unlaminar air-flow conditions through a narrow lumen. Air sacs are particularly prone to contamination because they have an air flow regime that favors particle deposition, no available macrophages to remove foreign items, and an epithelial surface that is nearly devoid of a mucociliary transport mechanism [155]. In this primary nidus, thermophilic spores find ideal temperature and aeration conditions to break their dormancy, germinate, and produce multicellular hyphae [53]. Mycelial development causes tissue necrosis and incites a strong host reaction than can be granulomatous and/or infiltrative, depending on the immune status of the bird [156]. It relies on a mixed inflammatory response that involves the recruitment of macrophages, multinucleated giant cells, and heterophils. These cells form within the lung parenchyma as characteristic granulomas around a necrotic center containing radiating hyphae which may be encapsulated by an outer layer of fibrous tissue [88] or constitute plaques lining air sac membranes or airways. In vitro, macrophages of rock pigeons demonstrate a limited capacity to kill phagocytized spores but inhibit their germination unless the spores are not too numerous. The fact that intracellular germination and subsequent cell death may occur following multiple spore ingestion may explain the limited efficiency of this first line in cases of overwhelming exposure to spores [148]. Heterophil granulocytes of the second line kill non-ingested hyphae by oxidative and non-oxidative mechanisms. When the immune response is less effective, infiltrative types of tissue reactions include exudative cellular inflammation with giant cells, macrophages, heterophils, and lymphocytes. In that case, the fungus can spread from the respiratory system via the circulatory system through pneumatized bones or by simple extension from the air sac wall to contiguous organs or cavities. Hematogenous or lymphatic dissemination of fungal elements is allowed by hyphal penetration of the lung blood vessels and by means of macrophages carrying viable spores. Under appropriate aerobic conditions, fungal asexual reproduction within air sacs is a common feature associated with plaques becoming velvety and changing color depending on the *Aspergillus* species involved [156].

Many species of *Aspergillus* are able to synthesize mycotoxins [7]. The exact role of these secondary metabolites in the development of aspergillosis remains unclear. The ergoline alkaloid fumigaclavine A has been shown to be produced by *A. fumigatus* during the course of clinical aspergillosis in the lungs of falcons (*Falco* sp.) [156]. In turkeys, high concentrations of gliotoxin, a mycotoxin with an immunosuppressive effect, have been detected in tissues obtained from birds with spontaneous airsacculitis [157] or in the lungs of birds experimentally inoculated with *A. fumigatus* [157]. Finally, turkey blood peripheral lymphocytes, when exposed to high levels of gliotoxin, either died or exhibited a lower lymphoblastogenic response [158]. This review does not address mycotoxicosis due to the ingestion of mycotoxins (aflatoxins in particular) which have already been shown to be responsible for high mortality rates in wild avifauna [159] in connection with moldy grain or contaminated feeders [160,161].

Using discriminant molecular tools, a constant and very high polymorphism of *Aspergillus fumigatus* isolated either from the environment or internal organs of both healthy and diseased birds has been demonstrated in several studies [5,162]. However, the origin of this remarkable variability, the role of sexual reproduction in its occurrence, and its putative pathological implications remain uncertain [53]. Polyclonal infections have already been reported in captive penguins and free-ranging white stork chicks [30,123].

Finally, it is still not clear why there is selective pressure for continued animal pathogenicity among fungi that are well adapted to abiotic environments.

## 7. Clinical Signs

Aspergillosis expression in birds may be reported during field outbreaks and the careful monitoring of its progression in diseased, captive birds or experimentally inoculated animals. Affected free-living birds are generally found moribund or dead. Acute aspergillosis usually presents with fairly non-specific signs such as lethargy, dullness, and ruffled feathers [27,88]. Loss of interest in food and anorexia are common observations, but pronounced weight loss is rather associated with chronic forms of the disease. Free-ranging birds may be reluctant to escape, walk with effort, or fly due to suffering from shot injuries and are therefore caught easily [58]. Polydipsia, polyuria, wing drop, stunting, and sudden death occur regularly. All of these signs are similar to those of lead poisoning [4,27]. Subtle non-specific first signs observed by falconers are a decrease in preening activity, a loss of ability to engage in prey hunting and even fly, and a failure to bathe [95,99,134]. More characteristic is the development of progressive and severe dyspnea with gasping, accelerated open-mouth breathing, tail-bobbing, and sometimes, a non-productive cough. Gurgle, rales, or wheezy sounds and a change in voice may be heard in cases of mycotic tracheitis [11,39,88,134,137,139,163]. Central nervous system involvement causes a loss of muscular coordination, twisting of the neck, and a head held in abnormal positions [12,27,44,89,99]. In controlled experiments of acute aspergillosis, respiratory signs (tachypnea, dyspnea with gasping, abdominal breathing), depression, anorexia, and ruffled feathers are common in inoculated (IT) starlings, pigeons, Japanese quails, and rock pigeons. Less frequent observations include greenish urates and vomiting [146,147,148,164].

An unusual presentation may affect organs other than the respiratory tract. Aspergillosis rhinitis and sinusitis cause nasal discharge in parrots [102]. Abrams et al. [145] described a severe bilateral inflammation and dermatitis of the eyelids that progressively extended to the head in a falcon hybrid. *Aspergillus* infections of the internal chambers of the eye are not rare in parrots and cause epiphora, blepharospasm, photophobia, periorbital swelling, and corneal ulcers [29,144]. Abnormal limb movements and paralysis associated with spinal or perirenal lesions have been reported in game pheasants [165], a black palm cockatoo (*Probosciger aterrimus*) [166], and a bufflehead duckling (*Bucephala albeola*) [167]. Painful aspergillosis granulomas could generate feather damaging behavior and skin mutilation in parrots [168]. *Aspergillus* spp. is considered a secondary invader in extensive foot web necrotic lesions of unknown origin in wild swans caught for routine banding [169].

## 8. Gross Lesions

Birds that succumb to acute aspergillosis are generally in good flesh condition [170,171], while wasting (pectoral muscle atrophy and negligible subcutaneous and internal fat) and dehydration are common features of chronic infection [97,98,134,172]. Emaciation has been correlated with the progression of the disease in common loons sheltered for rehabilitation [140]. As infection generally develops following the inhalation of spores, typical primary lesions are found in the respiratory tract and may be restricted to this area [11,12,30,37,39,173]. Souza and Degernes [80] qualified the infection as mild when it affected only one organ (either the trachea, the lungs or the air sacs) and as severe when at least two locations were involved. Isolated syrinx or tracheal bifurcation involvement is not rare and may be life-threatening with possible asphyxiation [174]. Internal air-flow circulation means that lungs and posterior air sacs (thoracic and abdominal pairs) are infected more often than anterior ones. The severity and the degree of development of the disease determine both the morphology and extension of macroscopic lesions along the coelomic serosa [102]. Gross lesions, either alone or in association (invasive aspergillosis) with others, have been observed in/on the brain, kidneys, liver, spleen, intestine, testis, bones, pericardium, and aorta [13,35,36,44,88,103,128,166,167,172,175,176,177,178,179]. The initial phase is characterized by hemorrhagic and edematous lesions that progress to a granulomatous type of inflammation [140]. Macroscopic lesions consist of white-yellowish unique or multiple spherical nodules ranging from miliary (<1 mm in diameter) to large roughly spherical granulomas (>40 mm) involving serosae and parenchyma of one or multiple organs [44,131]. Parenchyma are either consolidated or scattered with dense granulomas. When coalescing in air sacs, these deposits, varying in size and shape, form cheesy caseous plaques covering the thickened membranes and even obstructing the entire lumina where fungal sporulation may occur, as evidenced by a grey-greenish to black cottony texture [96,156].

## 9. Histopathology

Hematoxylin-eosin staining is often completed with periodic acid-Schiff and/or Grocott, Gomori’s methenamine silver dyes in order to detect fungal elements in tissue sections [156,180]. The use of fluorescent staining with the optical brightener blankophor has proven to be a valuable tool [181].

**Figure 3:**
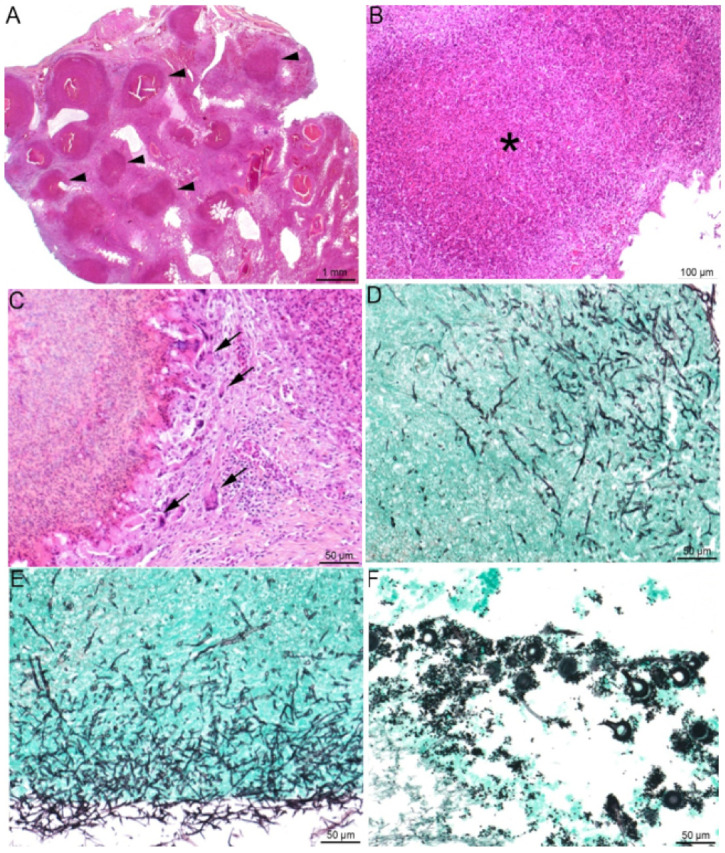
Lung histopathological lesions compatible with aspergillosis. (**A**–**E**) Lung of a knob-billed duck (*Sarkidiornis melanotos*). (**A**) Multifocal to coalescing heterophilic granulomas (black arrowheads), (**B**) displaying necrosis of heterophils and macrophages in their center (*) and (**C**) a peripheral rim of giant multinucleated cells (black arrowheads). In the granulomas (**D**) and the lung surface (**E**), the presence of invading thin (3- to 12 µm), non-pigmented (hyaline), septated hyphae with homogenous acute angle branching consistent with *Aspergillus* spp. (**F**) Lung of a red-crested pochard (*Netta rufina*) with numerous *Aspergillus* spp. conidial heads. (**A**–**C**): HES staining; (**D**–**F**): Gomori Grocott staining.

Microscopic examination of Canada geese victims of an aspergillosis outbreak led McDougle and Vaught [59] to describe three distinct types of lung infection: acute hemorrhagic pneumonia with few cells in the airways, a subacute form associated with caseous granulomas containing giant cells and radiating mycelia, and a chronic presentation characterized by extensive loss of normal tissue architecture following granuloma formation and hepatization. Based on histopathological differences, Cacciutello et al. [156] distinguished a deep nodular form of aspergillosis in non-aerated parenchyma and a superficial diffuse form in aerated tissue [171,182], although granulomas are frequently seen in the pulmonary parenchyma [97,140,178,181]. The classical structure of heterophilic granulomas consists of a necrotic center containing degenerate heterophils and radially arranged fungal elements surrounded by intact heterophils and a layer of epithelioid macrophages, multinucleated giant cells, and sparser lymphocytes or plasma cells [30] (Figure 3). In chronic forms, the effective host response can result in the formation of a fibrous layer circumscribing the granuloma. An *Aspergillus*-linked pneumonia without any gross lesion detection has been described following a systematic histopathological investigation in white storks. These pneumonia cases were characterized by multifocal poorly circumscribed aggregates of epithelioid macrophages and multinucleated giant cells surrounding filamentous fungus structures [30]. In the lungs, the infiltrative or diffuse forms of the disease induce hyperemia, micro-hemorrhages, a loss of epithelium lining in the bronchi, and its replacement with inflammatory exudate and cells (mostly degenerated heterophils and macrophages) extending to the peripheral parenchyma and pleura. The lung structure may be consequently replaced by multiple large areas of necrosis due to the coalescence of adjacent parabronchial foci [12,13,97,178]. The accumulation of various fungal elements (conidiophores, spores, hyphae), inflammatory exudate, and cells in and around airways are common features [28,44,171]. Fungal angioinvasion results in hemorrhages, vascular thrombosis, tissue infarction, and putative dissemination of *Aspergillus* to distant organs [23,172,182]. Air sac walls are diffusely thickened by infiltrates containing fibrin, heterophils, and macrophages, whereas their surface is colonized by mycelium with occasional conidial heads [17,98,131]. The presence of oxalate crystals in necrotized areas is an inconstant finding [17,23,95], but its frequency in avian aspergillosis could be underestimated, as demonstrated by Payne et al. [22]. More anecdotally, the ascospores, perithecia, and asci of *A. nidulans* have been documented in the lungs of an egret [25].

In several parrot species, microscopic lesions of the upper respiratory tract, possibly accompanied by malformation of the nostrils (with the presence of rhinoliths), beak, and cere, have been reported with hyphae filling the nasal cavity and paranasal sinuses, invading blood vessels, nerve bundles, turbinate cartilages, and nasal bones in severe cases [39,102]. In an enucleated eye of an amazon parrot, Hoppes et al. [144] described extensive, severe heterophilic, lymphoplasmacytic, and granulomatous keratitis, scleritis, and anterior uveitis. In the absence of respiratory lesions, the concentration of fungal elements in the cornea and, in less frequently, in the ocular chambers are suggestive of direct environmental contamination by contact.

## 10. Diagnostics

The recognition of aspergillosis in wild birds may be established either antemortem or postmortem [2,183]. Non-*Aspergillus* fungal species are able to produce similar lesions [30,180]. Furthermore, due to the ubiquitous nature of *Aspergillus* in the environment, positive cultivation from integument or respiratory tissues without any associated lesions may be frequent but should not be interpreted as a positive diagnosis of aspergillosis [3]. Therefore, a definitive diagnosis requires the identification of *Aspergillus* spp. from associated lesions [30]. Culture and isolation of the fungal agent for further characterization is considered the gold standard. In the absence of pathognomonic structures such as conidiophores, and since in vivo hyphal morphologies may overlap between *Aspergillus* fungi and multiple other fungi, histopathological observations of dichotomously branching hyaline hyphae with parallel walls on histopathological preparation may lead to false positives. In that case, immunohistochemistry with monoclonal or polyclonal antibodies is a powerful and accurate tool to identify in situ infections due to *Aspergillus* spp. or when mycological cultures remain negative [21,29,180,184].

Classical ante mortem diagnostic procedures include blood work, serology, and imaging [185]. However, ante mortem diagnosis of avian aspergillosis is much more challenging when cytological evaluation of clinical samples is not feasible. It is also problematic because extensive involvement of the respiratory tract can be present before clinical signs are apparent. Despite the presence of non-specific clinical signs, aspergillosis should be strongly suspected when debilitated birds suffering from respiratory distress are non-responsive to antibiotic treatment and when careful history may reveal the presence of underlying environmental or immunosuppressive factors [4,185].

In birds that are able to mount an appropriate immune response, blood work may reveal moderate to severe leukocytosis (20,000 to more than 100,000 cells per µL), heterophilia with a reactive left shift (toxic changes), monocytosis, and hyperproteinemia [4]. Plasma or serum protein electrophoresis can be used to obtain an overview of inflammatory changes. Both clinical and experimental data in falcons and psittacines indicate that an increase in β-globulins, hypoalbuminemia, a decreased albumin:globulin ratio (<0.5), and lower prealbumin values may be indicative of aspergillosis [105,186,187,188,189,190]. Desoubeaux et al. [191] combined a new biomarker with plasma protein electrophoresis in order to detect spontaneous cases of aspergillosis in a cohort of African penguins. Using 3-hydroxybutyrate, β-globulin, and α2-globulin measures in tandem resulted in a high level of specificity (>90%) and a negative predictive value (≥80%). The evaluation of two acute-phase proteins (haptoglobin and serum amyloid A) in falcons [190,192] and Japanese quails [193] gave contrasting results.

Two commercial ELISA kits have been used to detect two polysaccharidic components of the fungal cell wall: the galactomannan (GM) is relatively specific for *Aspergillus*, whereas (1-3)-β-glucan (BG) should be considered a panfungal test [194]. Different trials in various bird species showed low levels of sensitivity and poor correlations between the GM index and the disease status [187,188,190,191,195,196]. The BG assay appeared to be more suitable, although the results were also species-dependent with higher average concentrations seen in infected seabirds compared to raptors or companion birds [197]. PCR appears to be a very sensitive and cost-effective diagnostic tool which is still in its infancy in avian medicine. Mostly used for research purpose until recent years, PCR, generally based on 18S rRNA, allows the detection and identification of *Aspergillus* spp. isolated from field cases [21,30,97,123,130,198,199].

Serologic assays for the presence of anti-*Aspergillus* have been used in parrots, birds of prey, and penguins. However, the ubiquitous dispersal of *Aspergillus* spores and the inability of birds to mount an adequate immune response due to the animal’s severe disease state have resulted in false positives and false negatives, respectively, which greatly limit the diagnostic value of serology when used alone [40,41,99,186,187,190,200,201,202]. In a recent experimental model of acute aspergillosis in peregrine and peregrine x saker falcons, Wernery et al. [149] underlined the ability of anti-Afm1p (a highly immunogenic *A. fumigatus* galactomannoprotein) antibodies to discriminate inoculated birds from apparently healthy falcons by ELISA.

A combination of different diagnostic tools may overcome the intrinsic limitations of each test that are inherent to the variability of the host’s status and the disease severity. Hence, repeated testing can improve their levels of performance and can be used to evaluate disease progression and treatment success [201].

Radiography is part of the routine clinical examination of sick birds. However, the presence of radiologic signs indicates that birds are already in a late phase of the disease. Computer tomography and magnetic resonance imaging offer greater resolution and can highlight the invasive nature of the disease in birds. However none of these three imaging techniques can conclusively diagnose aspergillosis [203,204,205]. Endoscopy is an invasive procedure that readily enables veterinary practitioners to detect aspergillosis lesions (thickened air sac membranes, white-yellow, nodular, granulomatous, coalescing, plaque-like lesions, pigmented mold) and to collect samples (air sac lavages, biopsy, contact smears) for *Aspergillus* identification. Bronchoscopy is useful for the visualization of tracheal lesions and facilitates their removal [206].

## 11. Treatment

Treatment of avian aspergillosis, when possible, is not always successful because of the often advanced stage of the disease when the diagnosis is confirmed, the lack of pharmacokinetic data on antifungal drugs in most avian species, the failure of drugs to penetrate target tissues (especially encapsulated granulomatous lesions), and the frequent presence of concurrent diseases and/or immunosuppression [4,207]. Companion birds, raptors trained in falconry, and birds presented in zoos or to a lesser extent treated in wildlife rehabilitation centers can benefit from different therapeutic strategies. If a bird can tolerate anesthesia, the best way to overcome the disease is through topical therapy after surgical debridement via endoscopy of caseous material and granulomas, even in combination with early, aggressive, systemic, antifungal treatment [208,209]. Vacuum suction treatment proved to be effective for removing syringeal and tracheal mycotic obstructions detected by tracheoscopy in psittacines [174].

Historically, many protocols using different antifungal molecules or administration routes have been used as curative and even metaphylactic options in different species of wild birds. An oral solution of itraconazole (Fungitraxx, Floris, Vught, The Netherlands) was recently registered as the first antifungal product for ornamental birds in Europe. Given its broad antifungal spectrum and its fungicidal action on molds, voriconazole is increasingly being used to treat invasive aspergillosis in birds [207,209,210,211,212]. However, the empirical use of standard dosages to treat a great variety of bird species raises questions and underlines the need for more evidence-based data [207,209]. Recent research (Table 3) has focused on evaluating the bioavailability of the most promising molecules in different target species and more rarely on their therapeutic efficacy in experimental models of aspergillosis.

Both the formulation and the route of administration of antifungals should be carefully evaluated in targeted host species in order to reduce toxic effects and improve the long-term treatment of aspergillosis [213,214]. Effective management of captive birds, in particular, fragile individuals or birds at risk of immunosuppression (for example, following oil spill episodes), requires the minimization of handling stress. If subcutaneous implants of antifungal agents fail to reach targeted plasma concentrations [8,216], the nebulization of antifungal molecules could represent a promising technique that is applicable to groups of animals even prophylactically [216,217,218]. Some therapeutic protocols are summarized in Table 4.

In the framework of the one health context [219] and considering the limited number of drugs available to treat aspergillosis, the recent increase in antifungal resistance in human medicine should not be neglected by veterinarians and therefore should be carefully monitored [220,221]. The flight ability allows birds to travel great distances between cultivated fields that may be treated with fungicides. Consequently, birds might transfer *Aspergillus* isolates in this way, some of which could be resistant [222].

Using the CLSI method, the in vitro susceptibility of 59 avian *A. fumigatus* strains to amphotericin B, itraconazole, and voriconazole was determined. Four isolates showed acquired resistance to both itraconazole and voriconazole [223]. Twenty-two *Aspergillus* section *Fumigati* isolated from British captive penguins proved to be terbinafine- and voriconazole-sensitive, but all were resistant to itraconazole using minimum inhibitory concentrations cutoff values [224]. Investigation of antifungal susceptibility remains scarce in the field of wild avifauna and has been limited to newly identified *Aspergillus* species [33,34] or to a few drugs. All 18 clinical and 9 environmental *A. fumigatus* stricto sensu from a Californian rehabilitation center for seabirds were found to be sensitive to itraconazole [38]. Only one of 159 independent isolates from Germany was found to be azole (itraconazole and voriconazole) resistant [39]. In the framework of epidemiological surveys, more extensive screening could increase the detectability of resistant *Aspergillus fumigatus* isolates and even a multi-resistance pattern, as demonstrated in poultry contexts [225,226].

## 12. Prevention

Aspergillosis prevention measures are based on two main axes: controlling the level of exposure and minimizing stressors [4].

Risk management in a natural environment is limited to the first option when it is feasible. The abandonment of crop residues on the ground and rainy weather can promote the development of molds. Under unfavorable conditions, such as periods of snow or rain, crows and waterfowl can roam and ultimately land and feed on discarded moldy grains (corn) and silage [61,62,73]. Possible but limited solutions include burying, covering, or plowing under crop residues and reducing access to contaminated fields or piles by using audible scaring devices (pyrotechnics) to redirect birds to alternative feeding areas [2,27,242]. Grain used for avifauna baiting, trapping, or supplemental feeding programs should be properly stored and controlled. Fallow plots for wildlife should be regularly inspected. Keeping bird feeders and nest-boxes free of moldy substrates remains essential [2,88].

Captive conditions allow for finer control of the environment close to birds. As aspergillosis is not a contagious disease, multiple infections in a single enclosure involve common exposure rather than bird-to-bird spread [99]. It is useful to know the fungal loads to which animals can be exposed, especially in very sensitive species such as penguins or raptors. Air samples have been collected by sedimentation, filtration, or impaction in different environments housing birds. Bioimpactors, like the Air Strategie Bioimpactor, Surface Air Systems samplers, CIP 10-M, and Aerotech N6 (ex- Anderson N6) have been proven to be accurate tools for measuring the airborne *Aspergillus* concentration. The level of exposure when monitored by regular volumetric air sampling demonstrates important seasonal variations, a mitigating effect of low temperatures in cooled aviaries (Arctic and Antarctic species), and higher-risk micro-environments in multi-area structures [43,45,46,47,49,51,243]. Quantitative data are also useful to appreciate the effectiveness of filtration systems used in conventional air-handling systems to protect the most sensitive bird species, like penguins. High-Efficiency Particulate Air (HEPA) filters, although expensive and easily overloaded by the spore loads, are the best technical option but require meticulous maintenance [47]. In a Californian zoological park, among 22 variables tested, HEPA filters were shown to have the strongest effect (adjusted Odds Ratio = 4.33) on the reduction of *Aspergillus* prevalence in indoor sites [244]. Higher-than-average mortality rates due to aspergillosis can be associated with hot, humid climates, which predominate in tropical aviaries where many plants with a large bulk of litter and water pools are generally present. In brown kiwi nocturnal houses, variable concentrations of *A. fumigatus*, measured as CFU/g of wet material (soil, leaf litter), have been recovered with the highest counts following aspergillosis onset. Ground-dwelling species may be at greater risk of spore inhalation when foraging. Under stress, captive exotic birds may change their natural behaviors and spend more time on the ground foraging or hiding. By quantitatively estimating the background load levels of fungi, it becomes possible to identify inadequate litter management or storage that can promote *Aspergillus* growth. Regular cleaning and disinfection of the nest boxes, opening the canopy to increase the amount of sunlight reaching the floor of the aviaries, and ensuring proper ventilation help to reduce the risk of aspergillosis [42,43,47,48,85].

Animal facilities, transport crates, incubators, and hatchers should be adequately ventilated and cleaned and disinfected with antifungal agents (enilconazole and essential oils) before use to keep the infection pressure low [245,246]. Potential sources of spores, such as moldy litter materials and feed, should never be introduced. Minimizing plantings to limit plant and soil areas and choosing artificial rather than organic material for nests must be considered for penguin exhibits [2,46,49,119]. Finally, an indirect way of evaluating exposure to *Aspergillus* spp. can consist of repeated screening of antibodies, either in the serum or in the egg yolks, as has already been done in penguins, but this requires further research [40,41].

Improved animal husbandry practices minimize any stress in facilities. Birds suffering from aspergillosis can ward off the infection if it is not too severe and if global stress is minimized and the environmental quality is maximized. Broad and prolonged use of antibiotics or immunosuppressive drugs should be used with caution [107]. A 3-week prophylactic treatment with terbinafine or itraconazole in highly susceptible species of raptors is recommended by [247] in the following cases: newly captured or admitted individuals, following a change of management, extreme heat conditions, and even systematically in young reared gyrfalcons (3–120 days of age). Prophylactic protocols with itraconazole are also common in *Spheniscidae* held in zoo or rescued after oil spills [208,248]. In a Brazilian center [120], groups of penguins that received this antifungal prophylactically had 1.8 times fewer animals with aspergillosis when compared with non-treated birds (12.2% versus 22.9%).

Vaccination strategies have been attempted in birds but with inconsistent results [115,126,249,250].

## 13. Concluding Remarks

To go further and improve our knowledge of aspergillosis in wild birds, some data should be implemented more systematically:

As the susceptibility of the host to the disease seems to vary according to the avian species, it is necessary to identify the affected species precisely, especially in the event of an epizootic [74].

When grouped mortality occurs, possible sources should be sought as soon as possible by collecting adequate samples and investigating potential risk factors [74].

When gross findings are exuberant and promptly detected, institutions sometimes bypass histopathology or complementary diagnostic tests and conclude that “aspergillosis” was the cause of death, thus potentially underestimating the prevalence of other fungal pathogens [30,131,251]. The rigorous identification of a case of aspergillosis and a clinical isolate should be based exclusively on the association of an *Aspergillus* spp. with lesions [30,252].

A more systematic search for other putative etiological agents in the context of epizootics or analyses of mortality cohorts in particular, could make it possible to better understand the role of *Aspergillus* as an agent of primary or secondary infections [80].

Accurate identification of both common and cryptic *Aspergillus* species should be systematically performed in epidemiological studies. Molecular tools allowing simultaneous identification of mutations associated with a decreased sensitivity to azole antifungals such as the *cyp*51A gene are already available [183,221]. A systematic identification of all clinical isolates at the species level could be important to predict antifungal susceptibility or the clinical spectrum of new pathogenic species [253].

The constant difficulty of finding a single infection biomarker covering all species should prioritize the reorientation of the evaluation of these biomarkers on the species or taxa considered as priorities. Similarly, further targeted pharmacokinetic studies are necessary to improve the effectiveness of treatments on important species of birds of prey, penguins, and psittacines [210,252].

## Figures and Tables

**Table 1:** First descriptions of mycotic diseases in birds (Urbain and Guillot, 1938).

Year of Notification	Species	Description
1813	Scaup duck	“Mold or blue mucor” within the air sac
1815	Eurasian Jay	Parasitic thallus in air sacs, bronchi and lungs
1816	Mute swan	Green mold in the aerial cavities
1826	Stork	Mold lining the internal face of air sacs and within long bones
1827	Raven	Mold in the lungs
1833	Flamingo	Mold in the pulmonary cavities
1841	Eider	A fungus in air sacs, bronchi, basin and wing bones; later identified as “*Aspergillus glaucus*” by Robin
1841	Parakeet	Mycotic lesions in the lungs
1842	Falcon/Owl	Mycotic lesions in the bronchi and air sacs
1842	Goose/Cormorant/Penguin or Razorbill	Mycotic lesions
1842	Bullfinch	White mold (“*Aspergillus candidus*”) in the air sacs
1848	Golden plover	Mold in the air sacs later identified as “*Aspergillus glaucus*” by Robin (1853)
1853	Gull/Pheasant	“*Aspergillus nigrescens*” found in the lungs and the air sacs
1857	Golden eagle/Ostrich	Mycelium in the airways
1866	Parrot	Mold in the lungs
1866	Loon	Mold in the lungs
1871	Goshawk	Mold in the air sacs
1873	Duck	Mold in the airways
1875	Flamingo	“*Aspergillus dubius*” in the airways
1875	Great bustard	“*Aspergillus fumigatus*” in the lungs and air sacs
1880	Cardinal/Finches/Parrots	Aspergillosis
1883	Parrot	“*Aspergillus glaucus*” in the lungs and bronchi
1883	Flamingos	Aspergillosis
1885	Ostrich	“*Aspergillus fumigatus*” in the lungs and air sacs
1887	Swan	Aspergillosis
1887	Pheasant	Aspergillosis
1890	Ducks	Aspergillosis
1891	Canari	“*Aspergillus aviarius*” in the coastal pleura
1894	Swan	Aspergillosis

**Table 2:** Prevalence of aspergillosis in mortality surveys of different birds.

Species	Period	Location	Status ^a^	% of Aspergillosis (Nb Cases/Total)	Diagnosis ^b^	Reference
Common loon (*Gavia immer*)	1970–1975	USA	F	18% (34/190)	G/C	[109]
	1976–1991	USA	F	6% (13/222)	G/C/H	[77]
	1970–1994	USA	F	7% (31/434)	G/C/H	[110]
	1992–1995	Canada	F	16% (5/31)	G/D/H	[79]
	1979–1999	USA	F	31% (33/105)	Uns.	[78]
	1987–2000	USA	F	3% (14/522)	G/H	[72]
Mute swan (*Cygnus olor*)	1996	UK	F	14% (2/14)	G/H	[71]
Bewick’s swan (*Cygnus bewickii*) Mute swan (*Cygnus olor*) Whooper swan (*Cygnus cygnus*)	1951–1989	UK	F	5% (7/150) 4% (8/183) 4% (1/23)	G/H	[69]
Trumpeter swan (*Cygnus buccinator*) Tundra swan (*Cygnus columbianus*)	1986–1992	USA	F	21% (18/115) 10% (2/21)	G	[67]
Trumpeter swan (*Cygnus buccinator*) Tundra swan (*Cygnus columbianus*)	2000–2002	USA	F	17% (62/365) 5% (2/35)	G/D	[80]
Seabirds (Guillemot *Uria aalge*; Razorbill *Alca torda*; Herring gull *Larus argentatus*; Kittiwake *Rissa tridactyla*; Oystercatcher *Haematopus ostralegus,* among others)	1992–1995	Belgium	F	2.9% (6/241)	G/H	[111]
Herring gulls (*Larus argentatus*), other gulls	1981–1982	USA	F	31% (50/161)	G/C/H	[81]
White stork (*Ciconia ciconia*)	2007–2008	Germany	F	28% (22/101)	G/C/H/S (ITS-1)	[30]
Eurasian cranes (*Grus grus*)	1998–2008	Germany	F	4% (7/143)	G/C/H	[82]
Whooping crane (*Grus americana*)	1982–1995	USA	C	7% (7/103)	G/H	[70]
Bewick’s swan *Cygnus bewickii;* Whooper swan *C. cygnus;* Black swan *C. atratus*; Black-necked swan *C. melanocophyrus*; Trumpeter swan *C. buccinator*; Tundra swan C. *columbianus*	1951–1989	UK	C	6.6% (adults) 5% (juveniles) 2.6% (downies)	G/H	[112]
Seaducks (European Eider *Somateria mollissima;* Scoters *Melanitta*; Sawbills *Mergus*; Goldeneyes *Histrionicus, Clangula, Bucephala*)	1959–1976	USA	C	17% (adults) 31% (juveniles) 27% (downies)	Uns.	[113]
Shelducks (*Tadorna* sp.) Sheldgeese (*Cyanochen* sp., *Chloephaga* sp.)	1959–1976	USA	C	16% (adults) 5% (juveniles) 25% (adults) 33% (juveniles) 6% (downies)	Uns.	[113]
Perching ducks (Wood duck *Aix sponsa*; Hartlaub’s duck *Pteronetta hartlaubii*, among others)	1959–1980	USA	C	7.5% (adults) 8.5% (juveniles)	Uns.	[91]
Stiff-tailed ducks (Ruddy ducks *Oxyura* sp.; Musk ducks *Biziura* sp.; Black-headed ducks *Heteronetta* sp.; White-backed ducks *Thalassornis* sp.)	1959–1980	USA	C	2% (adults) 14% (juveniles)	Uns.	[114]
Northern geese (Canada geese *Branta canadensis;* Lesser white-front geese *Anser erythropus*…)	1959–1980	USA	C	4% (adults) 15% (juveniles)	Uns.	[92]
Falcons (saker falcons *Falco cherrug*; peregrine falcons *F. peregrinus*…)	1998–2001	Saudi Arabia	C	10% (13/131)	G/C/H	[15]
Gentoo penguins (*Pygoscelis papua)*	1964–1988	UK	C	41% (128/314)	Uns.	[115]
Magellanic penguins (*Spheniscus magellanicus)*	1986	USA	C	61% (23/38)	G/H	[116]
Magellanic penguins (*Spheniscus magellanicus)*	2008–2018	USA	C	27% (23/85)	G/H	[117]
Psittacine birds (parrots, macaws, cockatoos)	1998–2017	Canada	C	1.7% (32/1850)	H/Uns.	[118]
Magellanic penguins (*Spheniscus magellanicus)*	2004–2005	Brazil	R	42% (5/12)	G/D/C/H	[119]
Magellanic penguins (*Spheniscus magellanicus)*	2004–2009	Brazil	R	20% (66/327)	G/C/H	[120]
Bald eagle (*Haliaeetus leucocephalus*) Golden eagle (*Aquila chrysaetos*)	1975–2013	USA	R	1% (35/2980) 1% (15/1427)	Uns.	[100]
Black-browed Albatross *(Thalassarche melanophris)*	2015–2017	Brazil	R	14% (3/14)	G/C/H/S (*benA, calM*)	[35]

^a^ F = free-ranging birds; C: permanent captivity; R: transitory captivity or rehabilitation; ^b^ based on gross lesions (G); direct fungal examination (D); fungal culture (C); histopathology (H); gene(s) sequencing (S); unspecified (Uns.).

**Table 3:** Bioavailability and efficacy of different antifungal agents tested experimentally in several avian species.

Antifungal Agent	Status/Species	Administration Route	Dose	Aim	Main Conclusions	References
Amphotericin B (Liposomal)	Mallard ducks (Healthy)	Intratracheal nebulization (atomizer)	3 mg/kg (single)	PK	Target dose of 1 µg/g of lungs reached (up to 9 days) No toxic changes (histological examination)	[217]
Terbinafine hydrochloride	Shelduck (*Tadorna tadorna*) (Healthy)	Oral	60 mg/kg (single dose)	PK	No adverse effects Antifungal concentration remains above target doses for several hours	[227]
Terbinafine hydrochloride	African penguins (Healthy)	Oral	3/7/15 mg/kg (single dose) 15 mg/kg sid 4 days	PK	15 mg/kg per day oral dose = putative treatment Slow elimination and tissue accumulation	[228]
Terbinafine hydrochloride	Red-tailed hawks (Healthy)	Oral	15/30/60 mg/kg (single dose)	PK	A dose of 22 mg/kg SID may be a potential treatment option to treat aspergillosis in raptors	[228]
Terbinafine hydrochloride	Hispanolian amazons (Healthy)	Oral	60 mg/kg (single)	PK	No adverse effect Putative treatment of aspergillosis	[229]
Terbinafine hydrochloride	Hispanolian amazons (Healthy)	Nebulization (15 min)	1 mg/mL solution	PK	Plasma concentration above the target dose up to 4 hr	[218]
Itraconazole (Itrafungol)	African penguins (Healthy)	Oral	20 mg/kg (single dose)	PK	Putative cost effective treatment	[230]
Itraconazole (Itrafungol)	Lesser flamingos (*Phoeniconaias minor*) (Healthy)	Oral	10 mg/kg (single dose)	PK	Plasma drug concentration > 0.5 µg/mL maintained for at least 24 h after a single dose	[231]
Itraconazole (Sporanox/powder)	Humboldt penguins	Oral (in a fish)	6/12 mg/kg SID/BID 14 days	PK	8.5 mg/kg BID or 20 mg/kg SID of commercial capsule may provide adequate steady-state therapeutic blood levels	[232]
Itraconazole (nanoparticules)	Japanese quail (Infected)	Nebulization (30 min)	4%/10% suspension SID 6 days	EAA	10% nanosuspension is well tolerated and alleviates acute aspergillosis	[219]
Itraconazole (nanostructured lipid carries)	Falcon (*Falco* sp.) (Healthy)	Nebulization (15 min) (nanonebulizer)	NS	-	No toxic effects on A594 cells Penetrates deeply into the respiratory tract (lungs and air sacs (gammascintigraphy)	[233]
Voriconazole	Falcons (*Falco* sp.) (Healthy vs. diseased)	Oral (crop gavage or incorporated into meat)	12.5 mg/kg BID 7/14 days	PK/ET	High interindividuality of voriconazole/no adverse effects Administration in meat is effective and avoids tress	[234]
Voriconazole	African penguins (Healthy)	Oral	5 mg/kg (single dose) 5 mg/kg SID	PK	Effective for the treatment of aspergillosis Potential toxicity due to drug accumulation	[235]
Voriconazole	Magellanic penguins (Healthy)	Oral (in a herring)	2.5/5 mg/kg (single dose)	PK	Above the target dose for least 24 h following the highest dose	[214]
Voriconazole	Hispanolian amazons (Healthy)	Oral	12/24 mg/kg (single dose) 18 mg/kg QID 11 days	PK	Decrease in plasma concentration following administration of multiple doses requiring adjustment	[236]
Voriconazole	Red-tailed hawks (Healthy)	Oral (gavage)	10 mg/kg (single dose) 10 mg/kg BID 14 days	PK	More frequent dosing (up to QID) may be necessary to maintain target concentration during prolonged therapy	[237]
Voriconazole	Falcons (*Falco* sp.) (Healthy)	Intramuscular injection	12.5 mg/kg (single dose)	PK	Target plasma concentration (> 1 µg/ml) maintained 16 to 20 h without clinical side effects	[215]
Voriconazole	Mallard ducks (Healthy)	Intravenous injection or oral (liquid/non liquid)	10 mg/kg (single dose) 10/20/40 mg/kg (single dose) 20 mg/kg SID 21 days	PK	No overt/histological signs of toxicity A dosing interval of at least 8–12 h at a dose of 20 mg/kg may be required	[238]
Voriconazole	Japanese quail (Infected)	Oral	20/40 mg/kg SID	PK/EAA	Prolonged survival and less fungal burden in the lungs with the highest dose. No necrotic lesions (histopathology	[239]
Voriconazole	Rock pigeon (Infected)	Oral	10 mg/kg BID 20 mg/kg SID	EAA	Reduction of clinical signs and *A. fumigatus* elimination at 10 mg/kg BID	[240]

BID: twice a day; EAA: experimental acute aspergillosis; ET: empirical treatment on birds with spontaneous aspergillosis; NS: not specified; PK: pharmacokinetic studies; QID: four times per day; SC: subcutaneous; SID: once per day.

**Table 4:** Antifungal treatments recommended for avian aspergillosis [4,99,241].

Avian Taxon	Antifungal Agent	Dose and Administration Route
Gamebirds	Itraconazole	10 mg/kg orally SID or BID
	Terbinafine	15 mg/kg orally BID
Parrots	Amphotericin B	Nebulization 1 mg/kg diluted to 1 mL with sterile water BID or TID
	Itraconazole	5–10 mg/kg orally SID or BID
		2.5–5 mg/kg orally SID in Grey parrot
	Voriconazole	12–18 mg/kg orally BID
Raptors	Itraconazole	10 mg/kg orally BID for 60 days
		5–10 mg/kg orally BID for 5 days then SID for 60-90 days
	Terbinafine	10–15 mg/kg orally BID for 6-8 weeks
	Voriconazole	10–18 mg/kg orally BID for 60 days
		12.5 mg/kg orally BID in falcons
Seabirds	Itraconazole	10–20 mg/kg orally SID
Waterfowl	Amphotericin B	Nebulization 12.5 mg diluted with 2.5 mL sterile water SID for 7 days
		7.5 mg/kg intratracheally TID
		3.25 mg/kg intravenously (in fluids) over 24 h
	Itraconazole	5–10 mg/kg orally SID for 4-8 weeks

BID: twice per day; TID: three times per day; SID: once per day.
